# 4-Bromo-2-meth­oxy-6-(1-phenyl-1*H*-benzimidazol-2-yl)phenol

**DOI:** 10.1107/S1600536812007313

**Published:** 2012-02-24

**Authors:** Shunsheng Zhao, Xiangrong Liu, Xingqiang Lü, Weixu Feng

**Affiliations:** a College of Chemistry and Chemical Engineering, Xi’an University of Science and Technology, Xi’an 710054, Shaanxi, People’s Republic of China; b College of Chemical Engineering, Northwest University, Xi’an 710069, Shaanxi, People’s Republic of China

## Abstract

The title compound, C_20_H_15_BrN_2_O_2_, crystallized with three independent molecules in the asymmetric unit. Intramolecular O—H⋯N hydrogen bonds induce coplanarity of the substituted benzene ring and the benzimidazole ring, with mean deviations from the planes of 0.0931 (10), 0.0448 (10) and 0.0083 (11) Å in the three mol­ecules.

## Related literature

For the properties and applications of similar compounds and their complexes, see: Piguet *et al.* (1993 ▶); Yang *et al.* (2006 ▶).
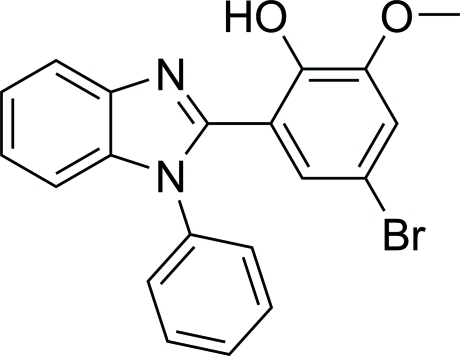


## Experimental

### 

#### Crystal data

C_20_H_15_BrN_2_O_2_
*M* *_r_* = 395.25
Triclinic, 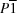

*a* = 12.377 (2) Å
*b* = 13.201 (2) Å
*c* = 17.474 (3) Å
α = 87.812 (3)°
β = 89.045 (3)°
γ = 64.652 (2)°
*V* = 2578.2 (8) Å^3^
*Z* = 6
Mo *K*α radiation
μ = 2.41 mm^−1^
*T* = 296 K
0.50 × 0.30 × 0.20 mm

#### Data collection

Bruker SMART 1K CCD diffractometer
Absorption correction: multi-scan (*SADABS*; Sheldrick, 2004 ▶) *T*_min_ = 0.379, *T*_max_ = 0.645
12738 measured reflections
8707 independent reflections
4152 reflections with *I* > 2σ(*I*)
*R*_int_ = 0.049

#### Refinement

*R*[*F* ^2^ > 2σ(*F* ^2^)] = 0.059
*wR*(*F* ^2^) = 0.178
*S* = 0.91
8707 reflections
679 parameters
H-atom parameters constrained
Δρ_max_ = 0.79 e Å^−3^
Δρ_min_ = −0.46 e Å^−3^

### 

Data collection: *SMART* (Bruker, 2001 ▶); cell refinement: *SAINT* (Bruker, 2001 ▶); data reduction: *SAINT*; program(s) used to solve structure: *SHELXS97* (Sheldrick, 2008 ▶); program(s) used to refine structure: *SHELXL97* (Sheldrick, 2008 ▶); molecular graphics: *SHELXTL* (Sheldrick, 2008 ▶); software used to prepare material for publication: *SHELXTL* and local programs.

## Supplementary Material

Crystal structure: contains datablock(s) I, global. DOI: 10.1107/S1600536812007313/fy2036sup1.cif

Structure factors: contains datablock(s) I. DOI: 10.1107/S1600536812007313/fy2036Isup2.hkl

Supplementary material file. DOI: 10.1107/S1600536812007313/fy2036Isup3.cml

Additional supplementary materials:  crystallographic information; 3D view; checkCIF report

## Figures and Tables

**Table 1:** Hydrogen-bond geometry (Å, °)

*D*—H⋯*A*	*D*—H	H⋯*A*	*D*⋯*A*	*D*—H⋯*A*
O2—H2*B*⋯N2	0.82	1.84	2.563 (6)	146
O4—H4*A*⋯N4	0.82	1.79	2.521 (7)	147
O6—H6*B*⋯N6	0.82	1.81	2.539 (7)	148

**Fig. 1.**
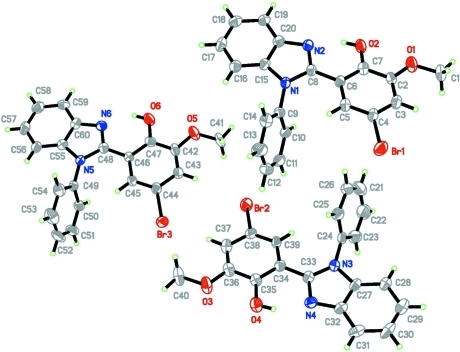
Molecular structure of (I), with atom labels and 30% probability displacement ellipsoids for non-H atoms.

**Fig. 2.**
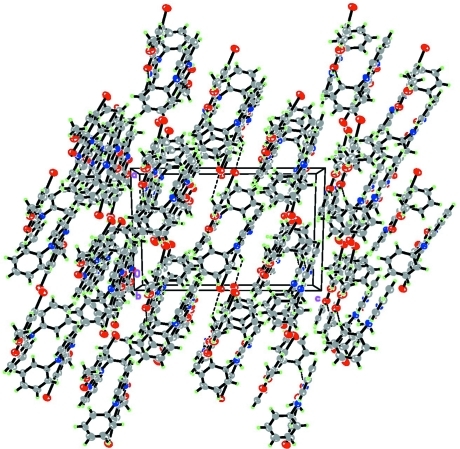
Crystal packing of (I), viewed from the b axis direction, showing the assembly of molecules connected by stacking interaction.

## supplementary crystallographic information

### Comment

The title compound contains three coordination sites (Fig. 1) and could form chelate compounds with several metals (Piguet *et al.*, 1993). A crystal packing diagram is shown in Fig. 2. The synthesis and structure of the title compound have not been reported previously, however, the synthesis and structure of a similar benzimidazole ligand and its 3d-4f bimetallic complex were described by Yang *et al.* (2006). Yang *et al.* (2006) also indicated that benzimidazole ligands could be synthesized from the reaction of substituted benzaldehyde and phenylene diamine.

### Experimental

The solution of 5-bromo-2-hydroxy-3-methoxybenzaldehyde (231.5 mg, 1.0 mmol) in ethanol (15 ml) was added to the solution of *N*-phenyl-1,2-phenylenediamine (184 mg, 1.0 mmol) in ethanol (10 ml) at room temperature. The mixture was stirred for 1 h before being heated to reflux, and was kept refluxing for another 2 h. Then it was cooled down to room temperature. The single crystal of the title compound suitble for X-ray diffraction was obtained by slow evaporation of the ethanolic solution.

### Refinement

H atoms bonded to O atoms were located in a diference map and were refined as idealised rotating hydroxyl groups, with O—H = 0.82 Å and *U*_iso_(H) = 1.2 U_eq_(O). Other H atoms were positioned geometrically and refined using a riding model with C—H = 0.95–0.99 Å and with *U*_iso_(H) = 1.2 (1.5 for methyl groups) times *U*_eq_(C).

### Crystal data

**Table:**

C_20_H_15_BrN_2_O_2_	*V* = 2578.2 (8) Å^3^
*M**_r_* = 395.25	*Z* = 6
Triclinic, *P*1	*F*(000) = 1200
Hall symbol: -P 1	*D*_x_ = 1.527 Mg m^−^^3^
*a* = 12.377 (2) Å	Mo *K*α radiation, λ = 0.71073 Å
*b* = 13.201 (2) Å	µ = 2.41 mm^−^^1^
*c* = 17.474 (3) Å	*T* = 296 K
α = 87.812 (3)°	Block, brown
β = 89.045 (3)°	0.50 × 0.30 × 0.20 mm
γ = 64.652 (2)°	

### Data collection

**Table:**

Bruker SMART 1K CCD diffractometer	8707 independent reflections
Radiation source: fine-focus sealed tube	4152 reflections with *I* > 2σ(*I*)
Graphite monochromator	*R*_int_ = 0.049
thin–slice ω scans	θ_max_ = 24.8°, θ_min_ = 1.8°
Absorption correction: multi-scan (*SADABS*; Sheldrick, 2004)	*h* = −13→14
*T*_min_ = 0.379, *T*_max_ = 0.645	*k* = −10→15
12738 measured reflections	*l* = −20→19

### Refinement

**Table:**

Refinement on *F*^2^	Primary atom site location: structure-invariant direct methods
Least-squares matrix: full	Secondary atom site location: difference Fourier map
*R*[*F*^2^ > 2σ(*F*^2^)] = 0.059	Hydrogen site location: inferred from neighbouring sites
*wR*(*F*^2^) = 0.178	H-atom parameters constrained
*S* = 0.91	*w* = 1/[σ^2^(*F*_o_^2^) + (0.0831*P*)^2^] where *P* = (*F*_o_^2^ + 2*F*_c_^2^)/3
8707 reflections	(Δ/σ)_max_ = 0.001
679 parameters	Δρ_max_ = 0.79 e Å^−^^3^
0 restraints	Δρ_min_ = −0.46 e Å^−^^3^

### Special details

**Table:**

Geometry. All e.s.d.'s (except the e.s.d. in the dihedral angle between two l.s. planes) are estimated using the full covariance matrix. The cell e.s.d.'s are taken into account individually in the estimation of e.s.d.'s in distances, angles and torsion angles; correlations between e.s.d.'s in cell parameters are only used when they are defined by crystal symmetry. An approximate (isotropic) treatment of cell e.s.d.'s is used for estimating e.s.d.'s involving l.s. planes.
Refinement. Refinement of *F*^2^ against ALL reflections. The weighted *R*-factor *wR* and goodness of fit *S* are based on *F*^2^, conventional *R*-factors *R* are based on *F*, with *F* set to zero for negative *F*^2^. The threshold expression of *F*^2^ > σ(*F*^2^) is used only for calculating *R*-factors(gt) *etc*. and is not relevant to the choice of reflections for refinement. *R*-factors based on *F*^2^ are statistically about twice as large as those based on *F*, and *R*-factors based on ALL data will be even larger.

### Fractional atomic coordinates and isotropic or equivalent isotropic displacement parameters (Å^2^)

**Table:**

	*x*	*y*	*z*	*U*_iso_*/*U*_eq_	
Br1	0.00077 (7)	0.68515 (7)	0.46124 (5)	0.0743 (3)	
N1	0.3704 (4)	0.7994 (4)	0.5502 (3)	0.0440 (13)	
N2	0.4927 (4)	0.6447 (4)	0.6175 (3)	0.0476 (13)	
O2	0.4881 (4)	0.4542 (3)	0.6025 (3)	0.0622 (13)	
H2B	0.5188	0.4973	0.6090	0.075*	
O1	0.3765 (4)	0.3364 (4)	0.5695 (3)	0.0715 (14)	
C5	0.2137 (6)	0.6783 (5)	0.5214 (4)	0.0519 (17)	
H5A	0.1769	0.7555	0.5125	0.062*	
C8	0.3980 (5)	0.6896 (5)	0.5723 (4)	0.0454 (16)	
C6	0.3297 (5)	0.6264 (5)	0.5534 (3)	0.0444 (16)	
C3	0.2053 (6)	0.5009 (6)	0.5188 (4)	0.0582 (19)	
H3A	0.1635	0.4595	0.5066	0.070*	
C4	0.1566 (6)	0.6153 (6)	0.5038 (4)	0.0548 (18)	
C9	0.2947 (6)	0.8670 (6)	0.4882 (4)	0.0522 (17)	
C11	0.2550 (7)	0.8968 (7)	0.3552 (4)	0.0620 (19)	
H11A	0.2728	0.8739	0.3050	0.074*	
C15	0.4578 (6)	0.8244 (5)	0.5833 (4)	0.0495 (17)	
C7	0.3794 (6)	0.5111 (5)	0.5695 (4)	0.0517 (17)	
C20	0.5338 (6)	0.7285 (5)	0.6244 (4)	0.0512 (17)	
C19	0.6288 (6)	0.7276 (6)	0.6638 (4)	0.0555 (18)	
H19A	0.6793	0.6639	0.6918	0.067*	
C2	0.3174 (6)	0.4486 (5)	0.5524 (4)	0.0523 (17)	
C18	0.6481 (6)	0.8231 (7)	0.6612 (4)	0.066 (2)	
H18A	0.7118	0.8242	0.6881	0.079*	
C10	0.3262 (6)	0.8306 (6)	0.4142 (4)	0.0483 (17)	
H10A	0.3929	0.7642	0.4047	0.058*	
C16	0.4755 (6)	0.9209 (6)	0.5796 (4)	0.0550 (18)	
H16A	0.4243	0.9849	0.5521	0.066*	
C14	0.1983 (6)	0.9658 (6)	0.5030 (4)	0.0613 (19)	
H14A	0.1796	0.9890	0.5530	0.074*	
C1	0.3150 (6)	0.2693 (6)	0.5557 (4)	0.075 (2)	
H1A	0.3650	0.1926	0.5701	0.113*	
H1B	0.2957	0.2746	0.5022	0.113*	
H1C	0.2427	0.2953	0.5853	0.113*	
C17	0.5733 (7)	0.9178 (6)	0.6188 (4)	0.069 (2)	
H17A	0.5893	0.9805	0.6166	0.083*	
C12	0.1596 (7)	0.9946 (7)	0.3693 (5)	0.079 (2)	
H12A	0.1137	1.0382	0.3285	0.094*	
Br2	0.37428 (7)	0.96530 (7)	0.17131 (5)	0.0786 (3)	
Br3	0.41421 (7)	1.27166 (7)	0.12526 (5)	0.0783 (3)	
N5	0.7530 (5)	1.4201 (4)	0.2285 (3)	0.0502 (14)	
N3	−0.0210 (4)	0.8637 (4)	0.1151 (3)	0.0498 (14)	
N4	−0.1573 (5)	1.0297 (4)	0.0687 (3)	0.0536 (14)	
N6	0.8865 (5)	1.2678 (4)	0.2909 (3)	0.0549 (14)	
O4	−0.1354 (4)	1.2109 (4)	0.0699 (3)	0.0670 (13)	
H4A	−0.1701	1.1704	0.0677	0.080*	
C48	0.7859 (6)	1.3098 (5)	0.2509 (4)	0.0477 (16)	
O5	0.7652 (4)	0.9565 (4)	0.2796 (3)	0.0768 (15)	
C38	0.2104 (6)	1.0387 (6)	0.1445 (4)	0.0572 (18)	
O6	0.8717 (4)	1.0819 (4)	0.3033 (3)	0.0702 (14)	
H6B	0.8970	1.1287	0.3112	0.084*	
C45	0.6128 (6)	1.2844 (5)	0.1953 (4)	0.0501 (17)	
H45A	0.5776	1.3591	0.1783	0.060*	
O3	−0.0138 (4)	1.3228 (4)	0.0921 (3)	0.0810 (16)	
C60	0.9217 (6)	1.3549 (6)	0.2946 (4)	0.0533 (18)	
C33	−0.0496 (5)	0.9752 (5)	0.0999 (3)	0.0464 (16)	
C24	0.0730 (6)	0.7788 (5)	0.1593 (4)	0.0489 (17)	
C55	0.8400 (5)	1.4492 (6)	0.2562 (4)	0.0493 (17)	
C47	0.7683 (6)	1.1307 (5)	0.2632 (4)	0.0523 (17)	
C46	0.7219 (5)	1.2415 (5)	0.2353 (4)	0.0468 (16)	
C32	−0.2039 (6)	0.9510 (6)	0.0633 (4)	0.0503 (17)	
C36	0.0439 (7)	1.2106 (6)	0.1089 (4)	0.0598 (19)	
C39	0.1457 (6)	0.9803 (5)	0.1350 (3)	0.0496 (17)	
H39A	0.1806	0.9030	0.1442	0.060*	
C34	0.0259 (6)	1.0347 (5)	0.1114 (3)	0.0482 (16)	
C27	−0.1203 (6)	0.8483 (6)	0.0919 (4)	0.0503 (17)	
C44	0.5595 (6)	1.2167 (6)	0.1817 (4)	0.0521 (17)	
C56	0.8518 (6)	1.5487 (6)	0.2518 (4)	0.0595 (19)	
H56A	0.7946	1.6129	0.2277	0.071*	
C43	0.6057 (6)	1.1058 (6)	0.2094 (4)	0.0572 (18)	
H43A	0.5664	1.0613	0.1999	0.069*	
C50	0.6786 (6)	1.4793 (6)	0.0996 (4)	0.0554 (18)	
H50A	0.7460	1.4200	0.0810	0.067*	
C37	0.1614 (7)	1.1559 (6)	0.1327 (4)	0.067 (2)	
H37A	0.2068	1.1953	0.1408	0.080*	
C35	−0.0223 (6)	1.1496 (6)	0.0960 (4)	0.0516 (17)	
C28	−0.1407 (6)	0.7526 (6)	0.0947 (4)	0.0603 (19)	
H28A	−0.0829	0.6841	0.1131	0.072*	
C41	0.7079 (7)	0.8844 (6)	0.2677 (4)	0.078 (2)	
H41A	0.7553	0.8114	0.2903	0.117*	
H41B	0.6998	0.8781	0.2138	0.117*	
H41C	0.6302	0.9152	0.2911	0.117*	
C49	0.6635 (6)	1.4936 (5)	0.1769 (4)	0.0483 (17)	
C42	0.7079 (7)	1.0642 (6)	0.2500 (4)	0.0564 (18)	
C51	0.5944 (7)	1.5520 (7)	0.0495 (5)	0.073 (2)	
H51A	0.6036	1.5407	−0.0029	0.087*	
C59	1.0220 (6)	1.3553 (6)	0.3294 (4)	0.064 (2)	
H59A	1.0777	1.2920	0.3552	0.077*	
C25	0.0775 (7)	0.7905 (6)	0.2378 (4)	0.064 (2)	
H25A	0.0240	0.8546	0.2616	0.077*	
C26	0.1649 (8)	0.7031 (9)	0.2790 (5)	0.083 (3)	
H26A	0.1704	0.7079	0.3316	0.100*	
C57	0.9523 (7)	1.5480 (7)	0.2846 (4)	0.070 (2)	
H57A	0.9652	1.6124	0.2804	0.084*	
C52	0.4975 (8)	1.6407 (8)	0.0764 (6)	0.089 (3)	
H52A	0.4413	1.6905	0.0422	0.107*	
C54	0.5670 (7)	1.5831 (6)	0.2056 (5)	0.070 (2)	
H54A	0.5582	1.5944	0.2580	0.084*	
C29	−0.2508 (7)	0.7631 (7)	0.0689 (4)	0.075 (2)	
H29A	−0.2679	0.7010	0.0697	0.090*	
C23	0.1492 (7)	0.6847 (7)	0.1239 (5)	0.072 (2)	
H23A	0.1437	0.6786	0.0714	0.086*	
C40	0.0516 (7)	1.3885 (6)	0.1012 (5)	0.089 (3)	
H40A	0.0017	1.4655	0.0873	0.134*	
H40B	0.1209	1.3611	0.0688	0.134*	
H40C	0.0761	1.3830	0.1536	0.134*	
C31	−0.3133 (6)	0.9621 (7)	0.0389 (4)	0.066 (2)	
H31A	−0.3708	1.0311	0.0209	0.080*	
C58	1.0348 (7)	1.4544 (7)	0.3235 (4)	0.073 (2)	
H58A	1.1003	1.4582	0.3463	0.088*	
C30	−0.3361 (7)	0.8680 (8)	0.0415 (4)	0.078 (2)	
H30A	−0.4098	0.8744	0.0248	0.093*	
C22	0.2351 (8)	0.5983 (7)	0.1667 (7)	0.099 (3)	
H22A	0.2868	0.5331	0.1433	0.119*	
C21	0.2435 (8)	0.6093 (9)	0.2429 (7)	0.095 (3)	
H21A	0.3034	0.5524	0.2712	0.114*	
C53	0.4822 (7)	1.6570 (7)	0.1535 (6)	0.092 (3)	
H53A	0.4152	1.7173	0.1715	0.111*	
C13	0.1292 (7)	1.0308 (7)	0.4424 (6)	0.083 (3)	
H13A	0.0631	1.0980	0.4513	0.100*	

### Atomic displacement parameters (Å^2^)

**Table:**

	*U*^11^	*U*^22^	*U*^33^	*U*^12^	*U*^13^	*U*^23^
Br1	0.0632 (5)	0.0791 (6)	0.0886 (6)	−0.0375 (4)	−0.0162 (4)	−0.0036 (5)
N1	0.049 (3)	0.037 (3)	0.050 (3)	−0.022 (3)	−0.002 (3)	−0.002 (3)
N2	0.045 (3)	0.051 (3)	0.051 (4)	−0.025 (3)	−0.002 (3)	0.001 (3)
O2	0.062 (3)	0.042 (3)	0.082 (4)	−0.022 (2)	−0.006 (3)	0.008 (3)
O1	0.072 (3)	0.044 (3)	0.100 (4)	−0.026 (3)	0.000 (3)	0.001 (3)
C5	0.055 (4)	0.046 (4)	0.060 (5)	−0.027 (3)	0.006 (4)	−0.006 (3)
C8	0.049 (4)	0.039 (4)	0.050 (4)	−0.021 (3)	0.002 (3)	−0.006 (3)
C6	0.042 (4)	0.050 (4)	0.047 (4)	−0.025 (3)	0.004 (3)	−0.005 (3)
C3	0.068 (5)	0.061 (5)	0.059 (5)	−0.041 (4)	0.009 (4)	−0.013 (4)
C4	0.062 (4)	0.053 (5)	0.059 (5)	−0.034 (4)	0.005 (4)	−0.010 (4)
C9	0.051 (4)	0.049 (4)	0.060 (5)	−0.024 (4)	−0.015 (4)	0.008 (4)
C11	0.076 (5)	0.076 (6)	0.050 (5)	−0.047 (5)	−0.003 (4)	−0.001 (4)
C15	0.052 (4)	0.053 (4)	0.048 (4)	−0.027 (4)	0.000 (3)	−0.008 (4)
C7	0.046 (4)	0.046 (4)	0.062 (5)	−0.018 (3)	0.011 (4)	−0.008 (4)
C20	0.051 (4)	0.045 (4)	0.054 (5)	−0.016 (3)	0.000 (4)	−0.007 (4)
C19	0.051 (4)	0.063 (5)	0.052 (5)	−0.024 (4)	−0.009 (3)	0.000 (4)
C2	0.056 (4)	0.046 (4)	0.059 (5)	−0.026 (4)	0.002 (4)	0.004 (4)
C18	0.066 (5)	0.074 (5)	0.062 (5)	−0.033 (4)	−0.018 (4)	−0.001 (4)
C10	0.051 (4)	0.056 (4)	0.044 (4)	−0.029 (3)	0.000 (4)	−0.003 (4)
C16	0.059 (4)	0.057 (4)	0.053 (5)	−0.027 (4)	0.005 (4)	−0.006 (4)
C14	0.049 (4)	0.056 (5)	0.075 (6)	−0.018 (4)	0.004 (4)	−0.008 (4)
C1	0.082 (5)	0.057 (5)	0.099 (6)	−0.043 (4)	0.016 (5)	−0.006 (4)
C17	0.073 (5)	0.071 (5)	0.082 (6)	−0.048 (5)	0.001 (4)	−0.017 (5)
C12	0.076 (6)	0.066 (6)	0.092 (7)	−0.029 (5)	−0.038 (5)	0.020 (5)
Br2	0.0685 (5)	0.0817 (6)	0.1002 (7)	−0.0467 (5)	−0.0171 (5)	0.0125 (5)
Br3	0.0888 (6)	0.0847 (6)	0.0824 (6)	−0.0581 (5)	−0.0341 (5)	0.0248 (5)
N5	0.058 (3)	0.045 (3)	0.052 (4)	−0.025 (3)	−0.014 (3)	0.000 (3)
N3	0.054 (3)	0.046 (4)	0.053 (4)	−0.025 (3)	−0.004 (3)	−0.002 (3)
N4	0.055 (4)	0.055 (4)	0.050 (4)	−0.023 (3)	−0.005 (3)	−0.002 (3)
N6	0.049 (3)	0.056 (4)	0.061 (4)	−0.023 (3)	−0.013 (3)	0.006 (3)
O4	0.058 (3)	0.055 (3)	0.086 (4)	−0.022 (3)	0.008 (3)	0.001 (3)
C48	0.052 (4)	0.046 (4)	0.046 (4)	−0.021 (3)	0.011 (3)	−0.007 (3)
O5	0.081 (3)	0.049 (3)	0.099 (4)	−0.029 (3)	−0.002 (3)	0.017 (3)
C38	0.067 (5)	0.062 (5)	0.050 (5)	−0.034 (4)	−0.008 (4)	0.002 (4)
O6	0.064 (3)	0.052 (3)	0.091 (4)	−0.021 (3)	−0.014 (3)	0.017 (3)
C45	0.062 (4)	0.046 (4)	0.049 (4)	−0.030 (4)	−0.002 (4)	0.007 (3)
O3	0.079 (4)	0.048 (3)	0.118 (5)	−0.030 (3)	0.010 (3)	−0.004 (3)
C60	0.047 (4)	0.057 (5)	0.058 (5)	−0.024 (4)	0.006 (4)	−0.013 (4)
C33	0.046 (4)	0.053 (4)	0.043 (4)	−0.023 (3)	0.011 (3)	−0.008 (3)
C24	0.048 (4)	0.043 (4)	0.065 (5)	−0.028 (3)	0.000 (4)	−0.001 (4)
C55	0.044 (4)	0.056 (5)	0.047 (4)	−0.020 (4)	0.005 (3)	−0.009 (4)
C47	0.050 (4)	0.045 (4)	0.051 (4)	−0.011 (4)	0.000 (4)	0.000 (4)
C46	0.050 (4)	0.044 (4)	0.047 (4)	−0.021 (3)	−0.003 (3)	0.002 (3)
C32	0.045 (4)	0.067 (5)	0.040 (4)	−0.024 (4)	0.007 (3)	−0.012 (4)
C36	0.074 (5)	0.048 (5)	0.058 (5)	−0.027 (4)	0.019 (4)	−0.008 (4)
C39	0.057 (4)	0.049 (4)	0.053 (4)	−0.033 (4)	0.001 (3)	0.002 (3)
C34	0.059 (4)	0.052 (4)	0.037 (4)	−0.027 (4)	0.005 (3)	−0.006 (3)
C27	0.048 (4)	0.071 (5)	0.045 (4)	−0.037 (4)	0.007 (3)	−0.015 (4)
C44	0.056 (4)	0.056 (5)	0.051 (4)	−0.032 (4)	0.006 (3)	0.000 (4)
C56	0.060 (4)	0.054 (5)	0.070 (5)	−0.029 (4)	−0.009 (4)	−0.005 (4)
C43	0.070 (5)	0.049 (5)	0.058 (5)	−0.030 (4)	−0.001 (4)	−0.001 (4)
C50	0.057 (4)	0.059 (5)	0.055 (5)	−0.030 (4)	−0.003 (4)	0.005 (4)
C37	0.070 (5)	0.060 (5)	0.079 (6)	−0.036 (4)	0.005 (4)	−0.006 (4)
C35	0.054 (4)	0.048 (4)	0.051 (5)	−0.020 (4)	0.009 (3)	−0.006 (4)
C28	0.060 (4)	0.067 (5)	0.063 (5)	−0.036 (4)	−0.001 (4)	−0.010 (4)
C41	0.105 (6)	0.050 (5)	0.085 (6)	−0.040 (5)	0.015 (5)	0.013 (4)
C49	0.052 (4)	0.044 (4)	0.055 (5)	−0.027 (4)	−0.003 (4)	0.000 (4)
C42	0.073 (5)	0.047 (5)	0.056 (5)	−0.033 (4)	0.013 (4)	−0.002 (4)
C51	0.078 (6)	0.082 (6)	0.065 (6)	−0.041 (5)	−0.016 (5)	0.024 (5)
C59	0.057 (4)	0.072 (5)	0.060 (5)	−0.023 (4)	−0.016 (4)	0.006 (4)
C25	0.075 (5)	0.072 (5)	0.055 (5)	−0.040 (4)	−0.003 (4)	−0.001 (4)
C26	0.097 (7)	0.106 (7)	0.073 (6)	−0.070 (6)	−0.027 (6)	0.026 (6)
C57	0.078 (5)	0.074 (6)	0.081 (6)	−0.052 (5)	0.002 (5)	−0.012 (5)
C52	0.082 (7)	0.075 (6)	0.109 (8)	−0.036 (6)	−0.036 (6)	0.050 (6)
C54	0.071 (5)	0.064 (5)	0.074 (6)	−0.027 (5)	0.003 (5)	−0.004 (5)
C29	0.071 (5)	0.088 (6)	0.081 (6)	−0.047 (5)	0.010 (5)	−0.029 (5)
C23	0.072 (5)	0.068 (5)	0.073 (6)	−0.027 (5)	−0.006 (5)	−0.005 (5)
C40	0.105 (6)	0.055 (5)	0.118 (7)	−0.046 (5)	0.037 (6)	−0.012 (5)
C31	0.052 (4)	0.094 (6)	0.051 (5)	−0.029 (4)	−0.003 (4)	−0.013 (4)
C58	0.067 (5)	0.092 (6)	0.074 (6)	−0.044 (5)	−0.008 (4)	−0.007 (5)
C30	0.064 (5)	0.119 (7)	0.072 (6)	−0.059 (6)	0.007 (4)	−0.037 (6)
C22	0.086 (7)	0.067 (6)	0.138 (10)	−0.025 (5)	−0.027 (7)	0.005 (7)
C21	0.079 (6)	0.093 (8)	0.117 (9)	−0.044 (6)	−0.021 (7)	0.041 (7)
C53	0.065 (6)	0.075 (6)	0.125 (9)	−0.020 (5)	0.010 (6)	0.010 (6)
C13	0.064 (5)	0.071 (6)	0.099 (7)	−0.012 (4)	−0.023 (5)	0.000 (6)

### Geometric parameters (Å, º)

**Table:**

Br1—C4	1.896 (7)	C45—H45A	0.9300
N1—C8	1.380 (7)	O3—C36	1.363 (8)
N1—C15	1.398 (7)	O3—C40	1.431 (8)
N1—C9	1.446 (8)	C60—C55	1.379 (8)
N2—C8	1.322 (7)	C60—C59	1.393 (8)
N2—C20	1.410 (7)	C33—C34	1.475 (8)
O2—C7	1.355 (7)	C24—C23	1.361 (9)
O2—H2B	0.8200	C24—C25	1.390 (9)
O1—C2	1.366 (7)	C55—C56	1.381 (8)
O1—C1	1.423 (7)	C47—C46	1.394 (8)
C5—C4	1.347 (8)	C47—C42	1.403 (9)
C5—C6	1.413 (8)	C32—C31	1.372 (8)
C5—H5A	0.9300	C32—C27	1.387 (9)
C8—C6	1.469 (8)	C36—C37	1.382 (9)
C6—C7	1.395 (8)	C36—C35	1.398 (9)
C3—C4	1.381 (9)	C39—C34	1.403 (8)
C3—C2	1.387 (9)	C39—H39A	0.9300
C3—H3A	0.9300	C34—C35	1.389 (8)
C9—C14	1.369 (9)	C27—C28	1.389 (8)
C9—C10	1.388 (8)	C44—C43	1.394 (8)
C11—C12	1.353 (10)	C56—C57	1.375 (9)
C11—C10	1.380 (9)	C56—H56A	0.9300
C11—H11A	0.9300	C43—C42	1.345 (9)
C15—C16	1.382 (8)	C43—H43A	0.9300
C15—C20	1.392 (8)	C50—C51	1.372 (9)
C7—C2	1.388 (8)	C50—C49	1.370 (9)
C20—C19	1.368 (8)	C50—H50A	0.9300
C19—C18	1.380 (9)	C37—H37A	0.9300
C19—H19A	0.9300	C28—C29	1.390 (9)
C18—C17	1.389 (9)	C28—H28A	0.9300
C18—H18A	0.9300	C41—H41A	0.9600
C10—H10A	0.9300	C41—H41B	0.9600
C16—C17	1.386 (9)	C41—H41C	0.9600
C16—H16A	0.9300	C49—C54	1.376 (9)
C14—C13	1.385 (10)	C51—C52	1.360 (11)
C14—H14A	0.9300	C51—H51A	0.9300
C1—H1A	0.9600	C59—C58	1.384 (9)
C1—H1B	0.9600	C59—H59A	0.9300
C1—H1C	0.9600	C25—C26	1.383 (10)
C17—H17A	0.9300	C25—H25A	0.9300
C12—C13	1.371 (11)	C26—C21	1.373 (11)
C12—H12A	0.9300	C26—H26A	0.9300
Br2—C38	1.894 (7)	C57—C58	1.382 (10)
Br3—C44	1.903 (7)	C57—H57A	0.9300
N5—C48	1.376 (7)	C52—C53	1.371 (11)
N5—C55	1.388 (7)	C52—H52A	0.9300
N5—C49	1.424 (8)	C54—C53	1.403 (10)
N3—C33	1.375 (7)	C54—H54A	0.9300
N3—C27	1.397 (7)	C29—C30	1.405 (10)
N3—C24	1.434 (8)	C29—H29A	0.9300
N4—C33	1.328 (7)	C23—C22	1.383 (11)
N4—C32	1.395 (8)	C23—H23A	0.9300
N6—C48	1.324 (7)	C40—H40A	0.9600
N6—C60	1.397 (7)	C40—H40B	0.9600
O4—C35	1.358 (7)	C40—H40C	0.9600
O4—H4A	0.8200	C31—C30	1.386 (10)
C48—C46	1.468 (8)	C31—H31A	0.9300
O5—C42	1.373 (8)	C58—H58A	0.9300
O5—C41	1.432 (8)	C30—H30A	0.9300
C38—C39	1.344 (8)	C22—C21	1.355 (12)
C38—C37	1.407 (9)	C22—H22A	0.9300
O6—C47	1.353 (7)	C21—H21A	0.9300
O6—H6B	0.8200	C53—H53A	0.9300
C45—C44	1.347 (8)	C13—H13A	0.9300
C45—C46	1.407 (8)		
			
C8—N1—C15	105.8 (5)	C45—C46—C48	122.6 (6)
C8—N1—C9	129.1 (5)	C31—C32—C27	120.2 (7)
C15—N1—C9	122.5 (5)	C31—C32—N4	131.1 (7)
C8—N2—C20	105.6 (5)	C27—C32—N4	108.6 (5)
C7—O2—H2B	109.5	O3—C36—C37	124.5 (6)
C2—O1—C1	117.1 (5)	O3—C36—C35	115.2 (7)
C4—C5—C6	119.7 (6)	C37—C36—C35	120.1 (7)
C4—C5—H5A	120.1	C38—C39—C34	120.7 (6)
C6—C5—H5A	120.1	C38—C39—H39A	119.7
N2—C8—N1	112.9 (5)	C34—C39—H39A	119.7
N2—C8—C6	120.9 (6)	C35—C34—C39	118.1 (6)
N1—C8—C6	126.1 (6)	C35—C34—C33	118.7 (6)
C7—C6—C5	118.5 (6)	C39—C34—C33	123.2 (6)
C7—C6—C8	118.9 (6)	C32—C27—C28	122.5 (6)
C5—C6—C8	122.6 (6)	C32—C27—N3	107.3 (6)
C4—C3—C2	119.0 (6)	C28—C27—N3	130.2 (7)
C4—C3—H3A	120.5	C45—C44—C43	122.2 (6)
C2—C3—H3A	120.5	C45—C44—Br3	120.5 (5)
C5—C4—C3	122.3 (6)	C43—C44—Br3	117.3 (5)
C5—C4—Br1	119.6 (5)	C57—C56—C55	116.7 (7)
C3—C4—Br1	118.0 (5)	C57—C56—H56A	121.7
C14—C9—C10	121.9 (7)	C55—C56—H56A	121.7
C14—C9—N1	120.2 (7)	C42—C43—C44	119.1 (6)
C10—C9—N1	117.8 (6)	C42—C43—H43A	120.5
C12—C11—C10	120.9 (7)	C44—C43—H43A	120.5
C12—C11—H11A	119.5	C51—C50—C49	120.2 (7)
C10—C11—H11A	119.5	C51—C50—H50A	119.9
C16—C15—C20	122.0 (6)	C49—C50—H50A	119.9
C16—C15—N1	131.1 (6)	C36—C37—C38	118.0 (6)
C20—C15—N1	106.9 (5)	C36—C37—H37A	121.0
O2—C7—C2	116.4 (6)	C38—C37—H37A	121.0
O2—C7—C6	123.0 (6)	O4—C35—C34	123.6 (6)
C2—C7—C6	120.5 (6)	O4—C35—C36	115.4 (6)
C19—C20—C15	120.5 (6)	C34—C35—C36	120.9 (6)
C19—C20—N2	130.7 (6)	C29—C28—C27	117.5 (7)
C15—C20—N2	108.8 (5)	C29—C28—H28A	121.3
C20—C19—C18	118.6 (6)	C27—C28—H28A	121.3
C20—C19—H19A	120.7	O5—C41—H41A	109.5
C18—C19—H19A	120.7	O5—C41—H41B	109.5
O1—C2—C3	125.1 (6)	H41A—C41—H41B	109.5
O1—C2—C7	115.0 (6)	O5—C41—H41C	109.5
C3—C2—C7	119.9 (6)	H41A—C41—H41C	109.5
C19—C18—C17	120.6 (6)	H41B—C41—H41C	109.5
C19—C18—H18A	119.7	C50—C49—C54	121.0 (7)
C17—C18—H18A	119.7	C50—C49—N5	120.0 (6)
C11—C10—C9	117.5 (6)	C54—C49—N5	118.9 (7)
C11—C10—H10A	121.2	C43—C42—O5	125.8 (6)
C9—C10—H10A	121.2	C43—C42—C47	120.6 (7)
C15—C16—C17	116.7 (7)	O5—C42—C47	113.7 (6)
C15—C16—H16A	121.7	C52—C51—C50	120.1 (8)
C17—C16—H16A	121.7	C52—C51—H51A	120.0
C9—C14—C13	119.2 (7)	C50—C51—H51A	120.0
C9—C14—H14A	120.4	C58—C59—C60	116.7 (7)
C13—C14—H14A	120.4	C58—C59—H59A	121.6
O1—C1—H1A	109.5	C60—C59—H59A	121.6
O1—C1—H1B	109.5	C26—C25—C24	117.3 (7)
H1A—C1—H1B	109.5	C26—C25—H25A	121.3
O1—C1—H1C	109.5	C24—C25—H25A	121.3
H1A—C1—H1C	109.5	C21—C26—C25	120.6 (8)
H1B—C1—H1C	109.5	C21—C26—H26A	119.7
C16—C17—C18	121.6 (7)	C25—C26—H26A	119.7
C16—C17—H17A	119.2	C56—C57—C58	122.0 (7)
C18—C17—H17A	119.2	C56—C57—H57A	119.0
C11—C12—C13	121.4 (8)	C58—C57—H57A	119.0
C11—C12—H12A	119.3	C51—C52—C53	120.4 (8)
C13—C12—H12A	119.3	C51—C52—H52A	119.8
C48—N5—C55	106.6 (5)	C53—C52—H52A	119.8
C48—N5—C49	131.8 (5)	C49—C54—C53	118.1 (8)
C55—N5—C49	120.8 (5)	C49—C54—H54A	121.0
C33—N3—C27	105.5 (5)	C53—C54—H54A	121.0
C33—N3—C24	132.0 (5)	C28—C29—C30	119.7 (7)
C27—N3—C24	121.3 (5)	C28—C29—H29A	120.1
C33—N4—C32	106.1 (5)	C30—C29—H29A	120.1
C48—N6—C60	105.5 (5)	C24—C23—C22	119.3 (8)
C35—O4—H4A	109.5	C24—C23—H23A	120.3
N6—C48—N5	112.0 (6)	C22—C23—H23A	120.3
N6—C48—C46	121.0 (6)	O3—C40—H40A	109.5
N5—C48—C46	127.0 (6)	O3—C40—H40B	109.5
C42—O5—C41	116.3 (6)	H40A—C40—H40B	109.5
C39—C38—C37	122.0 (6)	O3—C40—H40C	109.5
C39—C38—Br2	121.0 (5)	H40A—C40—H40C	109.5
C37—C38—Br2	116.9 (5)	H40B—C40—H40C	109.5
C47—O6—H6B	109.5	C32—C31—C30	118.4 (7)
C44—C45—C46	119.7 (6)	C32—C31—H31A	120.8
C44—C45—H45A	120.1	C30—C31—H31A	120.8
C46—C45—H45A	120.1	C57—C58—C59	121.4 (7)
C36—O3—C40	117.5 (6)	C57—C58—H58A	119.3
C55—C60—C59	121.1 (6)	C59—C58—H58A	119.3
C55—C60—N6	109.7 (6)	C31—C30—C29	121.7 (7)
C59—C60—N6	129.2 (7)	C31—C30—H30A	119.2
N4—C33—N3	112.4 (5)	C29—C30—H30A	119.2
N4—C33—C34	119.8 (6)	C21—C22—C23	119.6 (9)
N3—C33—C34	127.7 (6)	C21—C22—H22A	120.2
C23—C24—C25	122.1 (7)	C23—C22—H22A	120.2
C23—C24—N3	118.4 (7)	C22—C21—C26	121.0 (9)
C25—C24—N3	119.3 (6)	C22—C21—H21A	119.5
C60—C55—C56	122.0 (6)	C26—C21—H21A	119.5
C60—C55—N5	106.2 (6)	C52—C53—C54	120.3 (8)
C56—C55—N5	131.7 (6)	C52—C53—H53A	119.8
O6—C47—C46	122.9 (6)	C54—C53—H53A	119.8
O6—C47—C42	116.9 (6)	C12—C13—C14	119.0 (7)
C46—C47—C42	120.1 (6)	C12—C13—H13A	120.5
C47—C46—C45	118.3 (6)	C14—C13—H13A	120.5
C47—C46—C48	119.1 (6)		

### Hydrogen-bond geometry (Å, º)

**Table:**

*D*—H···*A*	*D*—H	H···*A*	*D*···*A*	*D*—H···*A*
O2—H2*B*···N2	0.82	1.84	2.563 (6)	146
O4—H4*A*···N4	0.82	1.79	2.521 (7)	147
O6—H6*B*···N6	0.82	1.81	2.539 (7)	148
